# Portal vein thrombosis associated with psoriasis: a case report

**DOI:** 10.1186/s13104-015-1046-7

**Published:** 2015-03-18

**Authors:** Jevon M Yudhishdran, Rayno Navinan, Sivakumar Jeyalakshmy, Ashoka Ratnatilaka

**Affiliations:** Department of Medicine, National Hospital of Sri Lanka, Regent Street, Colombo, Sri Lanka; Department of Medicine, Colombo South Teaching Hospital, Colombo, Sri Lanka

**Keywords:** Psoriasis, Venous thrombosis, Portal vein thrombosis

## Abstract

**Background:**

Psoriasis is no longer viewed as an isolated dermatological ailment and instead is considered a systemic disease. The extension of this spectrum has heightened the known risk of morbidity and mortality due to the involvement of cardiovascular system and the risk of venous thrombosis. A number of cases have reported the increased occurrence of deep vein thrombosis and pulmonary embolism in the background of psoriasis, however portal vein thrombosis has not been reported to date. We report an index case of chronic portal vein thrombosis in a diagnosed patient with psoriasis.

**Case presentation:**

A 67-year-old South-Asian female previously diagnosed and treated for psoriasis presented with a four month history of abdominal pain associated with abdominal distension. Clinical examination revealed an enlarged spleen and free fluid in the abdomen. Imaging with ultrasonography and computed tomography of the abdomen revealed features compatible with chronic portal vein thrombosis with cavernous transformation.

**Conclusion:**

This case highlights the importance of having clinical awareness of occurrence of thrombosis in patients with psoriasis. Typical symptoms favoring thrombosis should prompt thorough investigation to exclude this rare yet possible complication in patients with psoriasis, including that of portal vein thrombosis. Prophylaxis with anticoagulation still lacks strength of evidence to be justified in psoriasis. The exact pathogenesis of venous thromboembolism in psoriasis is still unexplained and further studies are needed to clarify the causal association.

## Background

Psoriasis is an inflammatory disease affecting 2% of the population [1,2]. The disease is no longer considered one that solely afflicts the skin, and with the available mounting scientific evidence the true systemic nature of the disease is becoming apparent. The impact of psoriasis is so expansive it can involve the lungs, kidneys, cardiovascular system, and also be associated with other autoimmune conditions, metabolic syndrome and even malignancy [3]. The impact of the Th1 inflammatory defect in psoriasis extends beyond afflicting the skin and joints and involves the endothelium as well [4]. A cohort study conducted by Ahlehoff *et al.,* in Denmark demonstrated age- and severity dependent increase in risk of cardiovascular and all-cause mortality in psoriasis patients [5]. Furthermore, patients with psoriasis also tend to have a higher overall rate of venous thrombo-embolism (VTE) events than controls [6,7]. However data pertaining to this is limited, as is the understanding to its possible pathophysiological mechanisms. An extensive search of literature revealed only a few reported cases of VTE occurring with psoriasis, involving that of the inferior vena cava, deep veins of the lower limbs, pulmonary vasculature, cerebral venous sinus and the central retinal vein [8-11]. We report a unique case of portal vein thrombosis in a patient with psoriasis.

## Case presentation

A 67-year-old South-Asian female who had been previously diagnosed with plaque psoriasis for the last 5 years and on topical treatment with emollient agents, coal tar salicylic shampoo and betamethasone cream presented with a complaint of abdominal distension associated with abdominal pain of four months duration in the absence of altered bowel habits. Her history was otherwise unremarkable and she denied any alcohol consumption, history of blood transfusion or sexual promiscuity and any long term oral drug treatment for her psoriasis, including the use of indigenous medication. There was also no history of neonatal umbilical vein cannulation and she denied any abdominal surgery or trauma.

General examination revealed well defined psoriatic plaque lesions on the forearms, elbows and in the scalp associated with scaling. The nails demonstrated dystrophy with pitting. Clinically she was found to be pale, but she had no lymphadenopathy, stigmata of liver disease or ankle oedema. Abdominal examination revealed ascites with shifting dullness and a firm, non- tender spleen extending 4 cm below the costal margin. Cardiovascular, respiratory, and nervous system examinations were unremarkable.

Whole blood analysis revealed a mild anemia with a haemoglobin of 9.8 g/dl (11–18) with a mean corpuscular volume of 88 fl (80–100). The white cell count was normal at 6.7 × 10^9^/L (4–10) with 69% neutrophils and 29% lymphocytes, and the platelet count was reduced at 52× 10^9^/L (150–450). Blood picture mirrored the whole blood findings and demonstrated a bicytopenia with a normocytic normochromic anemia and a moderate thrombocytopenia. Liver functions were within normal reference range. Aspartate amino-transaminase was at 20 IU/L (10–35) and alanine amino-transaminase was at 36 IU/L (10–40) while alkaline phosphatase levels were normal at 200 IU/L. Total serum protein was normal with a serum albumin of 36 g/L (36–48). Coagulation profile was normal. Imaging of the abdomen with an ultrasound scan confirmed a moderately enlarged spleen with the presence of varices, and thrombosis of the portal vein with cavernous transformation. A contrast enhanced computed tomography of the abdomen demonstrated multiple tortuous veins at the porta-hepatis, with an enlarged spleen and abnormally dilated tortuous vessels draining into the left renal vein with a normal sized liver with normal architecture. An upper gastrointestinal endoscopy showed early oesophageal varices. The radiological diagnosis was that of chronic portal vein thrombosis with cavernous transformation and a spleno-renal shunt.

The erythrocyte sedimentation rate(ESR) was elevated at 58 mm (0–15) for the 1st hour, and the C-reactive protein(CRP) was minimally elevated at 8 mg/dl (<5). The anti-nuclear antibody titres were normal. Anti-phospholipid antibody screening demonstrated a strongly positive beta 2 glycoprotein level with negative lupus anticoagulant and anti-cardiolipin antibodies. The beta 2 glycoprotein level was repeated after three months and was found to be positive. Thrombophilic screening for inherited prothrombotic states demonstrated normal antithrombin III, activated protein-C, protein-S levels and an undetectable factor V Leiden mutation. Bone marrow biopsy revealed only a moderately hyper cellular marrow with active megakaryocytes and was otherwise normal. Ham test was negative and JAK 2 mutation was also absent. Screening for occult malignancy with upper and lower gastrointestinal endoscopy, imaging of the chest, and mammogram failed to yield any positive findings. Retroviral screening was negative.

## Discussion

Portal vein thrombosis (PVT) can be acute, recent or chronic. Chronic portal vein thrombosis develops in patients’ whose thrombosis does not spontaneously resolve and can result in formation of a chronic non-cavernous thrombosed portal vein or cavernous transformation, as in our patient. The pathogenesis for PVT can be due to cirrhosis, local inflammatory lesions, injury to the portal venous system, malignancy as well as various states potentiating thrombophilia [12]. Venous thrombosis complicating psoriasis has been recorded quite early in medical literature, though it’s true association with the predominantly skin disease was still disputed at that time by Bunch *et al*., [10]. However subsequent observational reports impressed upon the increased incidence of thromboembolic disease seen in psoriasis [13]. A prospective analysis by Lutsey *et al*., demonstrated that being diagnosed with psoriasis increased the risk of incident venous thrombosis and embolism by as much as 40% [14].

The pathogenesis is still uncertain, and varied hypothesis and contributing phenomenon have been queried and linked to the occurrence of VTE in psoriasis. The proposed hypothesis include inflammatory processes either through genetic modulation via chromosomal coding of proteins associated with inflammatory proteins or directly quantifiable levels of inflammatory markers such as C-reactive protein(CRP), inflammatory cytokines [14-16] as precipitating causes of thrombosis or the occurrence of eosinophilia in psoriasis favoring a pro-thrombotic state [17], and the change in homocysteine levels either due to the disease process or its treatment, causing endothelial and vascular injury and triggering modifications in coagulation through plasmin, thrombomodulin and platelet hyperactivity and activation resulting in thrombosis [6,11,15,18]. Risk factors that predispose to VTE in psoriasis are also varied and include, immobility either due to disease process or other causes [16,17], young age, poor response to treatment [16] and disease severity. But it is noted that even mild disease may be an adequate risk factor for VTE [14]. Drugs have been associated with causing thrombosis in psoriasis in the past and have resulted in their discontinuation such as azarabine, an effect again possibly intertwined with homocysteine levels [19,20], etanercept, an anti-tumour necrosis factor [21] and acitretin, a retinoid [22]. Efalizumab, an anti-monoclonal antibody which blocks T-cell functionality and infliximab a chimeric monoclonal antibody targeting TNF-α [8,23] were also thought to result in thrombosis when used in psoriasis. Methotrexate has been purported to possibly have a protective effect through its anti-inflammatory properties [24] though simultaneously it could also cause a thrombotic event due to its dysregulation of homocysteine levels [6]. Our patient had none of these risk factors, as her disease was mild and she was on topical treatment with emollients, steroid and coal tar shampoo. Her CRP was not elevated though her ESR was elevated. Her beta 2 glycoprotein was repeatedly positive and maybe a causative phenomenon as it could signify secondary anti-phospholipid syndrome(APS) to that of the autoimmune psoriatic disease, as the association is noted [25]. Cassano *et al*., queried the same association but their analysis failed to reveal significant findings, though one subject with psoriasis was found to have anti-phospholipid antibodies [26], but Saigal *et al*., states the occurrence of anti-phospholipid antibodies can be high as 28% in psoriatic patients [27]. The occurrence of positive anti-phospholipid antibodies can be incidental or can be an aetiological factor for thrombosis formation in our patient. But the moderately elevated ESR and clinical picture favored the background psoriatic disease as the cause and the low platelets was most likely due to splenic pooling of platelets than that of findings favoring anti-phospholipid syndrome.

In psoriatic patients, the occurrence of new onset typical but non-specific symptoms of venous thrombo-embolism such as chest pain, shortness of breath, oedema, sudden onset headache, vision impairment should raise the suspicion of possible VTE [11] as pulmonary embolism [13], inferior vena caval occlusion [10], deep vein thrombosis of lower limbs, cerebral venous thrombosis [9] and central retinal vein occlusion [8] could be the underlying causes for these symptoms. Similarly abdominal pain and swelling in a psoriatic patient could point toward the consideration of a rare yet probable occurrence of portal vein thrombosis as in our case report. Though psoriasis is now recognized to be associated with VTE it is still an uncommon occurrence, and this may have led to the delay in both presentation and diagnosis.

Treatment of venous thrombosis is through anticoagulation, however the possibility of increased risk in psoriasis raises the concern whether prophylactic utilization of warfarin is warranted [11]. The opinion for this is divided and there is still no clear consensus, but Lutsey *et al*., concluded as the overall incidence is low VTE preventive therapy is unjustifiable [14]. Utilization of other possible therapeutic agents such as methotrexate was clinically unwarranted in our patient. Additionally when considering its side effect profile and risk-benefit ratio in consideration to its association with causing VTE makes it unsuitable. Even in other commoner clinical situations such as systemic lupus erythematosus, anticoagulation in the presence of positive anti-phospholipid antibodies in isolation without thrombosis is not recommended. The lack of clear consensus and the rarity of the clinical situation with little or no guidance will probably result in the clinician concurring with the general agreement of choosing not to prophylactically treat the possible risk of VTE, though it could potentially be fatal. The question arises as to whether occurrence of thrombosis in our patient was due to the associated pathophysiological mechanisms associated with psoriasis or true secondary anti-phospholipid syndrome to that of psoriasis. Additionally there is no clear role for anticoagulation with warfarin in a patient who has already developed cavernous transformation following portal vein thrombosis as the risk of bleeding in presence of varices discourages its utilization. With life-long warfarin therapy as the alternate choice and in consideration of the risks, we chose to defer instituting anticoagulation as prophylactic treatment.

## Conclusion

Though the pathogenesis is not fully understood, the risk of thrombosis in patients with psoriasis should be appreciated. Clinicians should be wary of the different ways in which venous thromboembolism can present in psoriasis and should have a high index of clinical suspicion when patients present with typical symptoms. Role of anticoagulants in prophylaxis appears unwarranted due to risks, lack of strong clinical evidence and definitive guidance. Screening patients with psoriasis for additional risk factors that promote thrombosis should be considered. We report this as an index case of portal vein thrombosis in a patient with psoriasis with anti-phospholipid syndrome.

## Consent

Written informed consent was obtained from the patient for publication of this case report. Copies of the written consent is available for review by the Editor-in-Chief of this journal.
